# Hip Morphology in Mucolipidosis Type II

**DOI:** 10.3390/jcm9030728

**Published:** 2020-03-08

**Authors:** Luise Sophie Ammer, Esmeralda Oussoren, Nicole Maria Muschol, Sandra Pohl, Maria Estela Rubio-Gozalbo, René Santer, Ralf Stuecker, Eik Vettorazzi, Sandra Rafaela Breyer

**Affiliations:** 1 Department of Pediatrics, University Medical Center Hamburg-Eppendorf, 20246 Hamburg, Germany; l.ammer@uke.de (L.S.A.); muschol@uke.de (N.M.M.); r.santer@uke.de (R.S.); 2 International Center for Lysosomal Disorders, University Medical Center Hamburg-Eppendorf, 20246 Hamburg, Germany; ralf.stuecker@kinderkrankenhaus.net; 3 Department of Pediatrics, Center for Lysosomal and Metabolic Diseases, Erasmus University Medical Center, 3015 GE Rotterdam, The Netherlands; e.oussoren@erasmusmc.nl; 4 Department of Osteology and Biomechanics, University Medical Center Hamburg-Eppendorf, 20246 Hamburg, Germany; s.pohl@uke.de; 5 Department of Pediatrics and Clinical Genetics, Maastricht University Medical Center, 6211 LK Maastricht, The Netherlands; estela.rubio@mumc.nl; 6 Department of Pediatric Orthopedics, Children’s Hospital Altona, 22763 Hamburg, Germany; 7 Department of Orthopedics, University Medical Center Hamburg-Eppendorf, 20246 Hamburg, Germany; 8 Department of Medical Biometry and Epidemiology, University Medical Center Hamburg-Eppendorf, 20246 Hamburg, Germany; e.vettorazzi@uke.de

**Keywords:** mucolipidosis type II, MLII, ML intermediate, I-cell disease, hip, hip dysplasia, hip dislocation, cloaking, femoral bowing, ultrasound

## Abstract

Mucolipidosis type II (MLII) is a rare lysosomal storage disorder caused by defective trafficking of lysosomal enzymes. Severe skeletal manifestations are a hallmark of the disease including hip dysplasia. This study aims to describe hip morphology and the natural course of hip pathologies in MLII by systematic evaluation of plain radiographs, ultrasounds and magnetic resonance imaging (MRI). An international two-centered study was performed by retrospective chart review. All MLII patients with at least one pelvic radiograph were included. A total of 16 patients were followed over a mean of 3.5 years (range 0.2–10.7 years). Typical age-dependent radiographic signs identified were femoral cloaking (7/16), rickets/hyperparathyroidism-like changes (6/16) and constrictions of the supra-acetabular part of the os ilium (16/16) and the femoral neck (7/16). The course of acetabular and migration indexes (AI, MI) significantly increased in female patients. However, in the overall group, there was no relevant progression of acetabular dysplasia with a mean AI of 23.0 (range 5°–41°) and 23.7° (range 5°–40°) at the first and last assessments, respectively. Better knowledge on hip morphology in MLII could lead to earlier diagnosis, improved clinical management and enables assessment of effects of upcoming therapies on the skeletal system.

## 1. Introduction

Mucolipidosis type II and III (MLII/MLIII) are rare lysosomal storage disorders caused by a deficiency of *N*-acetylglucosamine-(GlcNAc) 1-phosphotransferase. This enzyme catalyzes the first step of tagging lysosomal enzymes with mannose 6-phosphate (M6P) recognition markers for their receptor-mediated transport to the lysosomes [1]. Hence, in MLII and III, absent or reduced GlcNAc-1-phosphotransferase activity causes defective trafficking of multiple lysosomal enzymes and consecutive accumulation of non-degraded macromolecules in dysfunctional lysosomes [2]. Mutations in the *GNPTAB* gene coding for the α/β-subunit of GlcNAc-1-phosphotransferase are causing MLII (MIM #252500) or ML III alpha/beta (MIM #252600), whereas ML III gamma (MIM #252505) is originating from a defective GlcNAc-1-phosphotransferase γ-subunit due to mutations in the *GNPTG* gene [3,4,5]. MLIII alpha/beta patients are presenting an attenuated phenotype of MLII [6]. With predominant skeletal symptoms, they are overall less affected. Individuals with a phenotype between MLII and MLIII are referred to as ML intermediate [7,8,9].

MLII was first described by Leroy and Demars in 1967 as I-cell disease [10]. The term “mucolipidosis” was introduced in 1970 by Spranger and Wiedemann to describe several conditions with features both of mucopolysaccharidoses (MPS) and sphingolipidoses [11]. MLII is a progressive multi-organ disease usually with prenatal clinical onset and fatal outcome within the first decade of life due to cardiopulmonary complications [2]. Clinical features of MLII patients such as craniofacial dysmorphia, progressive cardiac dysfunction, hepatosplenomegaly, skeletal deformities and neurocognitive retardation resemble Hurler syndrome (MPS IH) [12,13]. Among other symptoms, orthopedic pathologies are present at birth and may include thoracic deformity, kyphosis, deformed long bones, dislocated hips, clubfeet and joint contractures [6,9]. Patients with MLII present distinct radiographic patterns at different age periods. Vertebral body rounding, sacrococcygeal sclerosis, talocalcaneal stippling, periosteal cloaking, rickets/ hyperparathyroidism-like changes are found in infants [14]. These signs then convert into radiographic features also known in MPS patients and referred to as dysostosis multiplex. They involve an abnormal J-shaped sella turcica, paddle shaped ribs, anterior inferior beaking of vertebral bodies, flared iliac wings and hip dysplasia [6,14].

Hip pathologies, specifically acetabular dysplasia and hip dislocation, have been described in MLII before [7,9], but a detailed report of hip morphology and an investigation of the natural course of hip pathologies have not been performed so far. Better knowledge on the characteristic changes in MLII hips might enable earlier diagnosis of the disease and improve clinical management of MLII patients including prevention of false hip dysplasia treatment decisions. Furthermore, taking into account that MLII is yet untreatable, information on the natural course of the disease is particularly important to be able to assess effects of experimental and potentially upcoming therapies (e.g., hematopoietic stem cell therapy, anti-inflammatory drugs, gene therapy) on the skeletal system. This study aims to improve understanding of hip morphology and the natural course of hip joint development in patients with MLII by a systematic evaluation of plain radiographs, hip ultrasounds and magnetic resonance imaging (MRI).

## 2. Materials and Methods

### 2.1. Patients 

This international two-centered study was performed by retrospective chart review of MLII patients and carried out at the interdisciplinary outpatient clinic of the International Center for Lysosomal Disorders in Hamburg, Germany and the Center for Lysosomal and Metabolic Diseases of the Erasmus University Medical Center, Rotterdam, The Netherlands. Inclusion criteria were clinically and biochemically and/or molecular genetically confirmed diagnosis of MLII and at least one radiograph of the pelvis. As some patients presented a less severe phenotype than expected in classical MLII they were referred to as ML intermediate. Patients with ML intermediate still manifest severe multi-systemic symptoms. Therefore, they are here included among MLII patients. Patients with clinically and biochemically and/or molecular genetically confirmed diagnosis of MLIII were excluded from the study.

### 2.2. Imaging

#### 2.2.1. Radiography

**Figure 1:**
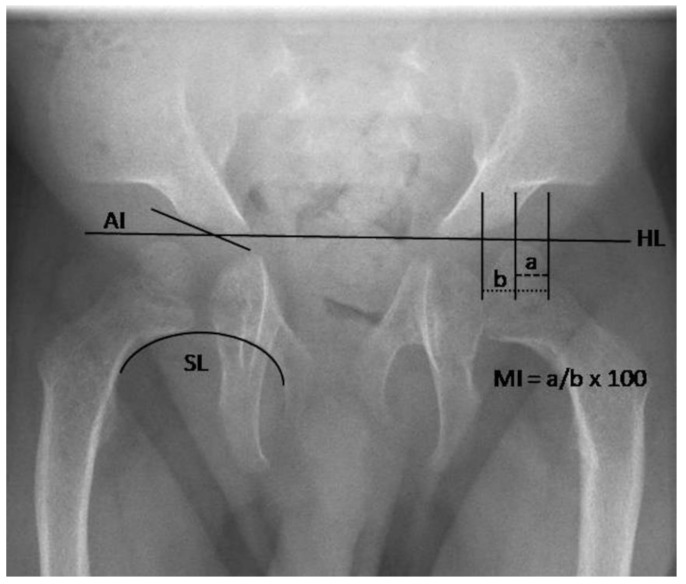
Quantitative radiographic assessments in MLII. Plain radiograph of a 2.8-year-old male patient. AI, acetabular index; SL, Shenton’s line; MI, Reimer’s migration index; HL, Hilgenreiner’s line.

Plain radiographs were digitally transferred and evaluated by one experienced pediatric orthopedic surgeon using Centricity PACS Universal Viewer (Version 5.0, GE Healthcare, Little Chalfont, United Kingdom). Radiographic evaluations were performed on supine anteroposterior (AP) pelvic radiographs. Hip morphology was analyzed quantitatively by: the acetabular index (AI) as described by Tönnis, the Reimer’s migration index (MI), the femoral neck-shaft angle and the Shenton’s line (Figure 1) [15,16,17]. The AI is formed by a horizontal line connecting both tri-radiate cartilages (Hilgenreiner line) and a second line, which extends along the acetabular roofs (Figure 1). The AI is age-, sex- and side-dependent and should become progressively shallower with age. Hip dysplasia was defined by the AI in correlation with reference data by Novais et al. [18]. The MI is the ratio of the uncovered femoral head part to the total femoral head width [16]. Therefore, it describes a relation between acetabular coverage and positioning of the femoral head. The reference range of a healthy population is 17–26% [19]. The anatomy of the femoral neck was described by the femoral neck-shaft angle. It was classified as physiological (angle within reference range) or as varus (angle below reference range) or valgus (angle above reference range) deformity [15]. The Shenton’s line was drawn as a curved line along the inferior border of the superior pubic ramus and the medial border of the femoral neck and subsequently classified as continuous or disrupted [20]. The following radiological findings were assessed qualitatively: rickets/hyperparathyroidism-like changes (subperiosteal or intracortical bone resorption with areas of cystic lucency and osteopenia), cloaking of the femur, femoral bowing, irregularities of the metaphysis or of the epiphysis of the proximal femur (flattening of the epiphysis, pathological ossification), the shape of the femoral neck and the configuration of the os ilium (constriction of the supra-acetabular portion of the pelvis, flared iliac wings). Patients without an ossified nucleus of the epiphysis were excluded from MI and femoral head assessments.

#### 2.2.2. Ultrasound

In Germany, hip ultrasound is routinely performed as part of regular medical assessments in newborns. Follow-up ultrasound examinations are common, for example, in high-risk patients for hip dysplasia. In the Netherlands, postnatal hip ultrasound is performed only in cases of clinical suspicion of hip dysplasia. Ultrasounds were performed with linear probes of 5.0 or 7.5 Megahertz (MHz). Hip dysplasia was classified according to Graf [21]. Original ultrasound images and their initial reports were reviewed.

#### 2.2.3. MRI

MRI images were used to analyze the position of the head of the femur in relation to the acetabular roof. It was defined as centered (maintained contact of the femoral head to the medial acetabular roof), lateralized (no medial contact of the femoral head with the acetabulum, but still a position below the acetabular roof) or dislocated (no acetabular coverage of the femoral head). The cartilage anlage of the acetabulum was described qualitatively. The rate of effusion was documented. Two different imaging systems were used: 1.5 T Avanto (Siemens AG Healthcare, Erlangen, Germany) and 3.0 T Philips Ingenia (Philips, Eindhoven, The Netherlands).

### 2.3. Statistics

Individual data was collected on both body sides and over time, thus introducing a cluster structure. We therefore used linear mixed models to model outcome variables with random intercepts and random slopes for patients. Time was treated as a continuous variable. To allow for deviations from linear time course polynomial spline trends were considered and interactions between time and sex, body side and ML type were initially included in the models. These models were simplified by removing insignificant interactions based on Likelihood Ratio tests (LRT) at a 5% level of significance. Estimated marginal means from final models were reported. All analyses were performed using the lmerTest-package in R 3.6.1 (R Core Team, Vienna, Austria) [22].

### 2.4. Ethics

A retrospective chart analysis of data acquired during routine visits was conducted in all study patients. Data was anonymized before analysis. Therefore, the need of ethic approval was waived.

## 3. Results

### 3.1. Patients

A total of 16 MLII patients were enrolled in the study (Table 1). Sex distribution was slightly imbalanced with nine males (56%) and seven females (44%). Eight patients had died by the time of chart review with a mean age of 6 years at death (range 0.3–11.8 years, SD 4.1). The mean age of patients alive at the last assessment (*n* = 8) was 3.8 years (range 0.7–11.2 years, SD 3.8). As four of the 16 patients presented a less severe phenotype than expected in classical MLII despite the presence of severe *GNPTAB* gene mutations in some of them, they were referred to as ML intermediate.

### 3.2. Imaging

#### 3.2.1. Radiography

Supine AP pelvic radiographs were performed in all patients. Fifteen patients had serial radiographs, adding up to 54 radiographs (108 hips) in 16 patients. In mean, radiographic assessment was done 3.4 times per patient (range 1–9 times, SD 2.1). The mean radiological follow-up time was 3.5 years (range 0.15–10.7 years, SD 3.2). The mean age at the first radiological assessment was 0.8 years (range 0–3.3 years, SD 1.0) and 4.7 years (range 0–11.2 years, SD 3.5) at the last assessment.

#### 3.2.2. Quantitative Assessment of Radiographs

**Figure 2:**
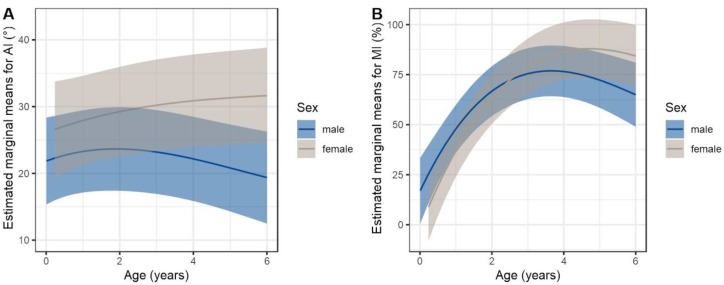
Estimated marginal means from final linear mixed model using spline functions for age. Trajectories differ significantly between sexes. (**A**) Acetabular index (AI) (LRT *p* = 0.016); (**B**) Migration index (MI) (LRT *p* = 0.036).

**Figure 3:**
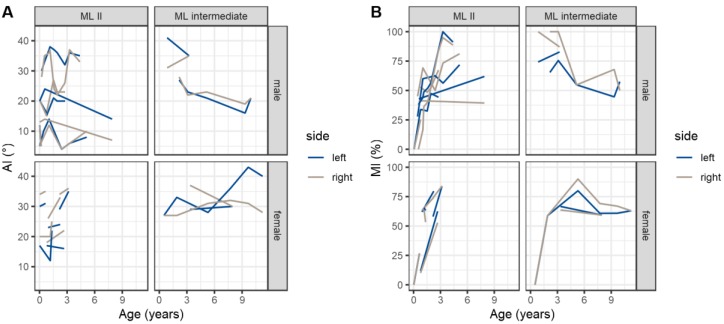
Comparison split by type and sex. Development of single left and right hips displayed by measurements over time. (**A**) acetabular index (AI); (**B**) migration index (MI).

**Figure 4:**
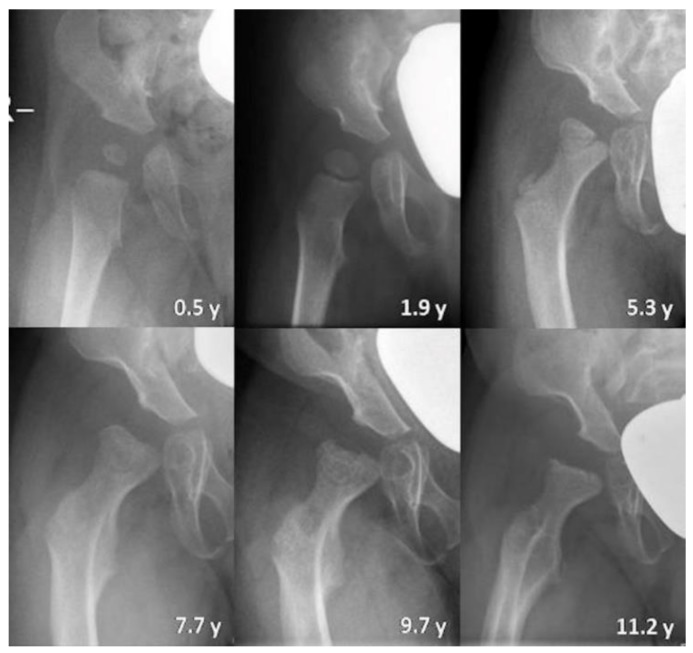
Hip development in a female ML intermediate patient (*n* 16) illustrated by plain radiography of the right hip at different years of age (y). Physiological measurements change into hip dysplasia with progressive pathological ossification of the femoral head and increasing constrictions of the supra-acetabular part of the os ilium and the femoral neck. The migration index increases but this does not result in high hip dislocation.

Overall, the mean AI at the first radiographic assessment was 23.0° (range 5°–41°, SD 8.7). At the first evaluation, 10/32 hips (6 patients, female:male 3:3, bilateral:unilateral 4:2) and, at last evaluation, 12/30 hips (seven patients, female:male 4:3, bilateral:unilateral 5:2) were classified as dysplastic. The AI remained stable with a mean AI of 23.7° (range 5–40, SD 10.4) at the last radiographic evaluation. Nevertheless, the course of AI (*p* = 0.016) and MI (*p* = 0.036) measurements differed significantly between sexes. (Figure 2 and Figure 3). Single AI values increased significantly in female patients from 4 years of age onwards. The mean MI at the first evaluation was 41.8% (range 0–100%, SD 30.8) and increased to up to 61.9% (range 25.3–91.2%, SD 19.6) at the last assessment. There was no significant difference between right and left hips concerning AI and MI measurements. Shenton’s line was disrupted in 8/32 hips at first and in 7/30 hips at the last assessment. None of the patients presented with a high dislocation of the femoral head (Figure 4).

The mean femoral neck-shaft angle at the first assessment was 146° with a great variability between individuals (range 102°–176°, SD 16.7). However, measurements remained nearly stable over time (mean 151°, range 120°–189°, SD 16.9). The distribution of all femoral neck angles was as follows: 6% physiological, 28% varus and 66% valgus.

No difference between MLII and ML intermediate was found concerning AI, MI or femoral neck-shaft angle measurements.

#### 3.2.3. Qualitative Assessment of Radiographs

**Figure 5:**
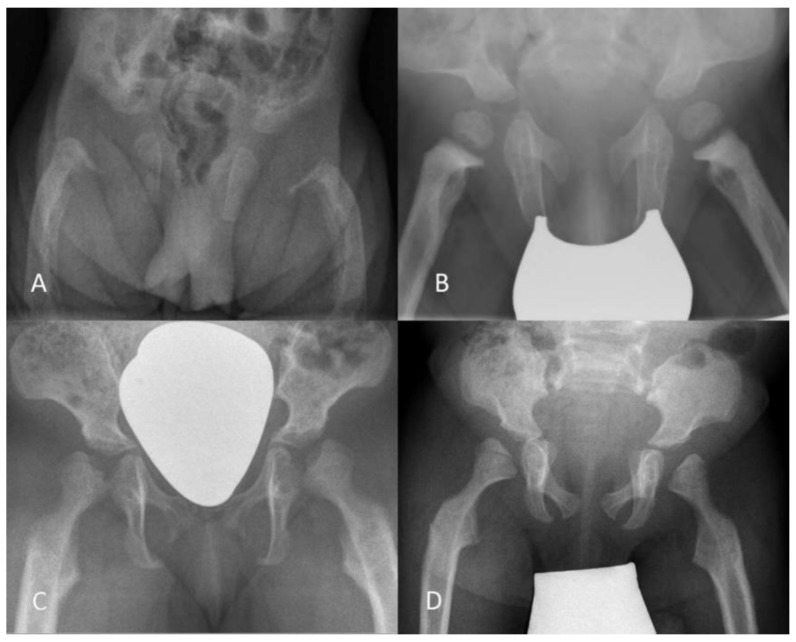
Qualitative radiographic assessment. (**A**) Rickets/hyperparathyroidism-like changes with metaphyseal irregularities, periosteal cloaking and femoral bowing in a male MLII patient at 1 week of age (*n* 11). (**B**) Coxa valga with widening of the growth plate of the proximal femur in a male MLII patient at 1 year of age (*n* 5). (**C**) Osteonecrosis–like changes of the femoral head, flared iliac wings in a female ML intermediate patient at 8 years of age (*n* 13). (**D**) Constriction of the femoral neck with medial flattening of the epiphysis in a male ML intermediate patient at 9.3 years of age (*n* 15). (**B–D**) Constrictions of the supra-acetabular part of the os ilium.

The qualitative radiologic assessment of hip morphology revealed the following frequent radiologic findings: rickets/hyperparathyroidism-like changes, periosteal cloaking, femoral bowing, widening of the growth plate of the proximal femur, osteonecrosis–like changes of the femoral head, flared iliac wings, constrictions of the supra-acetabular part of the os ilium and the femoral neck (Figure 5). Radiographic assessments in the first year of life existed in 12 patients and documented femoral cloaking in seven in 12 patients and rickets/hyperparathyroidism-like changes at the pelvis in six in 12 patients, both at a mean age of 0.26 months (range 0.1–0.7 months, SD 0.02). As temporary pathologies, femoral cloaking and rickets/hyperparathyroidism-like changes were no longer seen on radiographs after a mean of 8.9 months and 6.8 months, respectively. One patient had a spontaneous fracture of the proximal femur on the left side, which was diagnosed 8 days after birth. Femoral bowing was found in six in 16 patients at first evaluation and did not vanish over time. Moreover, it appeared in 3 more patients during growth (nine in 15). The development of the proximal femoral metaphysis was abnormal (irregularities in ossification, cupping or fraying) in nine in 16 patients at the first assessment. Metaphyseal changes appeared in three more patients during follow-ups and persisted in all affected patients (12/15) until the last assessment. A radio-transparent transverse metaphyseal band as a sign for osteolysis was seen in one female patient at 3 weeks of age. It was no longer seen on the following radiograph at 9.6 months of age.

The shape of the proximal epiphysis of the femur was asymmetrical with medial flattening of the femoral head in four in 13 patients at the first assessment. Deformation of the medial epiphysis increased over time: at the last radiological assessment, nine in 14 patients showed bilateral alterations of the shape of the femoral head. Among these patients, four in nine developed bilateral osteonecrosis-like disturbances of the femoral head. The os ilium was shaped abnormally in all patients at the last assessment with flaring of the iliac wings and constriction of the supra-acetabular portion. Bilateral constrictions of the femoral neck were seen in seven in 16 patients at the last assessment.

#### 3.2.4. Ultrasound

Ultrasound of the hips was performed in 10 patients (eight MLII, two ML intermediate, 20 hips), of whom five had serial ultrasound examinations (mean 1.8 times, range 1–5 times, SD 1.2), adding up to 19 ultrasounds.

**Figure 6:**
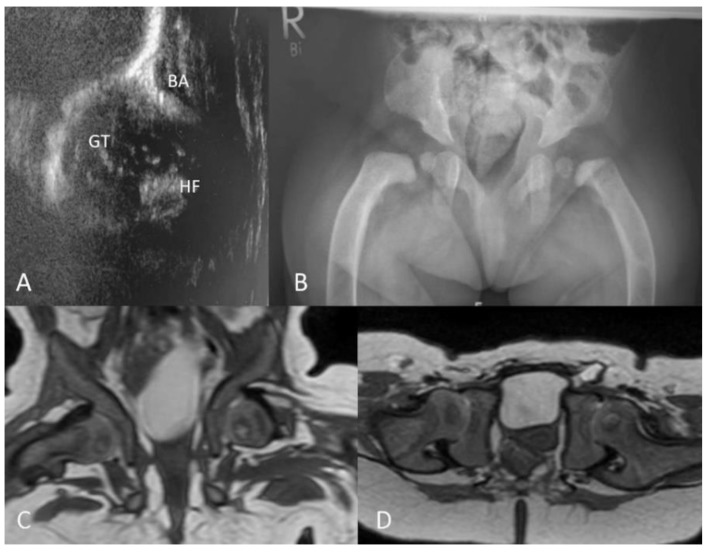
Hip morphology in a female MLII patient (*n* 3). (**A**) Ultrasound of the right hip at 1 week of age with an overriding greater trochanter; GT, greater trochanter; HF, head of the femur; BA, bony acetabulum. (**B**) Plain radiograph of the pelvis at 8 months of age presenting flared iliac wings, femoral bowing, an increased acetabular index, but a continuous Shenton’s line. (**C**,**D**) Magnetic resonance imaging (MRI) of the pelvis at 8 months of age, T2 weighted, in coronar and transverse views showing centered hip joints.

The mean age at the first ultrasound was 3.3 months (range 0.03–11.2 months, SD 3.6). Original ultrasound images with corresponding written reports were available for all patients. According to ultrasound reports, Graf’s classification distribution was as follows: IA: 25%, IB: 20%, IIA: 5%, IIB: 5%, IIC: 5%, IIIA: 20%, IIIB: 0%, IV: 20%. Hence, apparently, 10/20 were physiological, two in 20 critical and eight in 20 dislocated hips. A review of the original ultrasound images proved difficulties in anatomical interpretation; an overriding greater trochanter was interpreted incorrectly as a lateralized femoral head in four in 10 patients. In those four patients, dislocation of the hip joint was revised and excluded during the first 4 months of life by radiography (*n* = 2) or MRI (*n* = 2) (Figure 6). After all, only one hip joint was dislocated (Graf type IIIA) in the first month of life. Four patients were treated with an abduction-flexion hip orthosis, of whom two patients were falsely treated with an orthosis, because of misinterpretation of hip ultrasound.

#### 3.2.5. MRI

**Figure 7:**
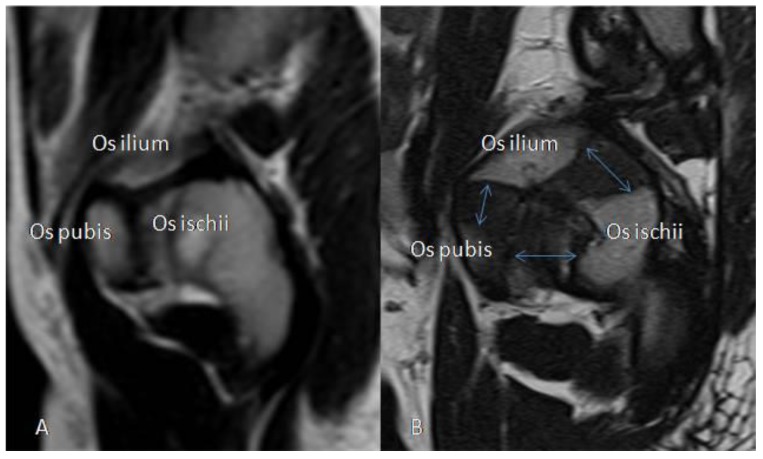
Magnetic resonance imaging (MRI) presentation of a widened tri-radiate cartilage of the acetabulum. MRI (T2 weighted, sagittal) of the tri-radiate cartilage of the left acetabulum (**A**) in a 5.6-year-old healthy male and (**B**) in a 6.7-year-old female ML intermediate patient (*n* 16) with widening of the cartilage (blue arrows).

MRI scans of the hips were performed in three patients (two MLII, one ML intermediate), of whom one had serial MRI examinations, adding up to four MRI scans. The mean age at the first MRI was 4.7 years (range 0.2–11.3 years, SD 5.31). At the first assessment, four in six hips were centered, two in six hips were lateralized, and none was dislocated. The patient with serial MRI scans showed no progression of primarily lateralized hips during 4.6 years of follow-up. There was no effusion in the hip joints. Sagittal planes existed for one female ML intermediate patient at 6.7 years of age showing a widening of the tri-radiate cartilage (Figure 7). In coronal planes, MRI displayed variable proportions of the lateral cartilaginous and labral coverage of the femoral head.

## 4. Discussion

The present study is the first report to describe hip morphology in MLII patients by a systematic evaluation of radiographs, hip ultrasounds and MRI scans. MLII was originally described as a Hurler variant and is often initially misdiagnosed as MPS IH [11]. The following radiological aspects are characteristic for MLII and can help to distinguish MLII from MPS: (1) typical bony changes in infancy: femoral cloaking and rickets/hyperparathyroidism-like changes; (2) acetabular dysplasia with a rather short than steep acetabular coverage; (3) constriction of the supra-acetabular part of the os ilium; and (4) constriction of the femoral neck.

Study patients were clinically subdivided in MLII and ML intermediate. Concerning hip morphology and progression of hip disease the two phenotypes showed no relevant differences. This result supports the decision to present MLII and ML intermediate patients as one group in this study. However, it might be biased by a limited follow-up time, considering that 50% of the patients are still alive at the time of chart review.

In the present study the rates of rickets/hyperparathyroidism-like changes and femoral cloaking in the first year of life were 50% and 58%, respectively. Lai et al. tracked 19 MLII patients from birth up to 2.5 years of age and found rickets/hyperparathyroidism-like changes in 33% and periosteal cloaking in 74% of the patients [14]. The age of disappearance ranged from 5 to 10 months. This observation also roughly equals up with the results of the present study, in which rickets/hyperparathyroidism-like changes and cloaking disappeared between 5 and 14 months of age.

Frequently described hip pathologies in MLII patients are dysplasia and dislocation, however, the current literature lacks exact pathological differentiation and classification [6,23,24,25,26]. Ocada et al. documented congenital dislocation of hip joints in 10/14 Japanese MLII patients without mentioning, how dislocation was diagnosed or whether both sides were affected [25]. It is known that the overall rate of hip dislocations in Japan is higher than in Western countries due to the Japanese tradition of swaddling [27]. This fact could lead to a higher rate of dislocated hips in MLII patients in Japan. It has been unclear, whether congenital hip dislocations in MLII patients are primarily congenital or occur as secondary pathologies, for example, due to impaired growth of the acetabulum, joint contractures or muscular hypotonia. In Germany, hip ultrasound is routinely performed as part of regular medical assessments in newborns. Hence, neonate hip ultrasounds were frequently done in patients of this study. Interestingly, hip ultrasound images could not confirm a high rate of congenital hip pathologies.

In healthy newborns, acetabular measurements often show sex differences with primarily higher AI values in females than in males. During growth, sex differences usually even out leading to a physiological AI decline [18]. In MLII, this decline is missing, which results in a significant sex difference concerning the course of MI and AI values. The reason for this remains unclear and it requires further research to determine to what extent the sex affects acetabular development in MLII.

An increasing AI can lead to insufficient acetabular coverage with migration of the femoral head and hence higher MI values. However, in MLII males with decreasing AI values, the MI increased in the first years of life. This can be caused by a general anatomical disproportion of acetabular and femoral structures in MLII patients. The tri-radiate cartilage of the acetabulum adheres to the three bony pelvic parts: ilium, ischium, and pubis and grows by endochondral ossification. In healthy children, it develops synchronized with the femoral head [28]. Severe disturbances of endochondral ossification are known in MLII [24,29,30]. This can explain hypoplastic iliac bones with constriction of the supra-acetabular portion and shortening of the acetabulum [6,11,31,32]. The acetabulum-femoral head disproportion could lead to the false assumption of increasing hip lateralization and therefore a growing rate of hip dislocations. However, the rate of disrupted Shenton’s lines remained stable. Notably, no patient showed a high dislocation of the femoral head at the last assessment. The results indicate that the pathology of MLII hip dysplasia is rather a short than steep acetabulum. This is in line with other studies reporting horizontal acetabular roofs in MLII patients [29,32].

The abnormal pelvic and acetabular shape in MLII patients might induce biomechanical forces on the femoral head leading to deformation, medial flattening and osteonecrosis-like changes of the epiphysis of the proximal femur (Figure 5). Both in the MLII mouse model and in MLII humans, the growth plate of the proximal femur is abnormally developed and widened due to lysosomal storage accumulation in chondrocytes [33,34]. This could make the femoral head even more susceptible to altered biomechanical forces.

Constriction of the femoral neck, which is present in almost half of the study patients (7/15) at the last assessment, has been described before in MLII, but not in MPS [6,24,35]. In healthy children, the three growth zones of the proximal femur, the longitudinal, trochanteric and isthmus plates, show proliferative activity throughout growth. Hereby, the femoral neck isthmus broadens the femoral neck laterally [28]. In MLII, a constricted femoral neck has been explained before by severe osteolysis [6]. However, growth disturbances in the three growth plates could also lead to a dysplastic, constricted neck and coxa vara or valga deformity of the proximal femur [36]. Considering the progress of femoral neck constriction over time, growth disturbances are more likely to be the cause.

Descriptions of varus and valgus deformities in MLII vary in the literature [6,12,26,37]. This might be, due to the fact, that measurements in patients with femoral bowing can be challenging. Reliable neck-shaft angle measurements depend on a standardized position of the lower extremities during radiography, which is difficult in MLII patients, due to joint contractures. Furthermore, they require 2-dimensional imaging with a second, lateral view of the proximal femur. Hence, the neck-shaft angle should be interpreted with caution.

Ultrasound findings in MLII patients were frequently misinterpreted as congenital hip dislocation, which prompted false treatment decisions. This may be due to a high riding greater trochanter as a result of femoral bowing or coxa vara, which can be mistaken for a lateralized femoral head. Furthermore, a horizontal acetabular bony roof and contractures of the hip joints can limit ultrasound visibility and lateral positioning of the patient for a standardized ultrasound examination. Ultrasounds therefore must also be interpreted with caution. Plain radiograph may be considered before introducing orthopedic measures, in order to help reduce unnecessary application of hip abduction braces.

This study has few limitations. The small number of patients is based on limited patient availability with MLII being an orphan disease. A limiting factor of the retrospective study design is the absence of a radiographic lateral frog-leg view of the hips, which is required for reliable analysis of the neck-shaft angle.

## 5. Conclusions

The present study proves distinct age-depended radiographic changes in MLII. Growth disturbances of os ilium parts lead to short acetabular coverage with increasing hip migration. However, although sex differences exist, the overall course of acetabular development does not result in rapid hip dysplasia progression or secondary high hip dislocations.

Hip ultrasounds performed in the neonatal period excluded high rates of congenital hip pathologies. An overriding greater trochanter was frequently misinterpreted as a dislocated femoral head, which prompted unnecessary orthopedic measures. Hence, hip ultrasounds should be interpreted with caution and radiographic assessment should be considered early. Better knowledge on hip morphology in MLII could lead to earlier diagnosis, improved clinical management and enable assessment of effects of upcoming therapies on the skeletal system. Further studies on the natural course of skeletal disease in a larger number of patients are needed.

## Figures and Tables

**Table 1:** Patient characteristics.

*n* (Sex)	Genotype *GNPTAB* Allele 1/allele 2	Phenotype	Age at Death (Years)	Age at First Radiograph (Years)	Age at Last Radiograph (Years)	Age at First Ultrasound (Month)	Age at First MRI (Years)
1 (F)	c.1001G>A/c.1001G>A	MLII	N/A	0.9	2.3	11.2	N/A
2 (F)	c.1337G>A/c.3098T>C	MLII	8.7	2.2	3.2	0.1	N/A
3 (F)	c.3335+1G>A/c.3335+1G>A	MLII	N/A	0.0	0.7	0.3	0.6
4 (M)	c.3503_3504del/c.1052dup	MLII	N/A	0.8	2.8	9.1	N/A
5 (M)	Unknown	MLII	9.4	0.0	5.1	1.6	0.2
6 (F)	Unknown	MLII	3.4	0.0	2.7	0.2	N/A
7 (M)	c.3503_3504del/c.3503_3504del	MLII	N/A	0.1	0.8	0.6	N/A
8 (M)	c.516dup/c.516dup	MLII	8.0	0.0	7.9	N/A	N/A
9 (M)	c.3503_3504del/c.3503_3504del	MLII	N/A	1.2	N/A	N/A	N/A
10 (M)	c.1090C>T/c.3091C>T	MLII	4.7	0.2	4.4	2.8	N/A
11 ^#^ (M)	c.3503_3504del/c.3503_3504del	MLII	0.3	0.0	0.2	N/A	N/A
12 ^#^ (F)	c.3503_3504del/c.3503_3504del	MLII	1.3	0.0	1.3	N/A	N/A
13 (F)	c.10A>C/c.10A>C	ML intermediate	N/A	3.3	8.0	N/A	N/A
14 (M)	Unknown	ML intermediate	N/A	0.8	3.2	0.0	N/A
15 (M)	c.10A>C/c.2502del	ML intermediate	11.8	2.2	10.0	N/A	6.7
16 (M)	c.344_345del/c.1022del	ML intermediate	N/A	0.5	11.2	0.9	N/A

F, female; M, male; N/A, not applicable; ML, mucolipidosis; MRI, magnetic resonance imaging; n, patient number; #, siblings.
